# The lived experience of a novel disruptive therapy in a group of men and boys with haemophilia A with inhibitors: Emi & Me

**DOI:** 10.1111/hex.13404

**Published:** 2021-12-08

**Authors:** Simon Fletcher, Kathryn Jenner, Michael Holland, Kate Khair

**Affiliations:** ^1^ Oxford Haemophilia and Thrombosis Centre Oxford University Hospitals NHS Foundation Trust Oxford UK; ^2^ Haemnet London UK

**Keywords:** burden of treatment, decision‐making, disruptive therapies, emicizumab, haemophilia, inhibitors

## Abstract

**Background:**

People with haemophilia A and inhibitors (PwHi) suffer more orthopaedic complications, bleeding and pain than those without inhibitors. The advent of emicizumab as a prophylactic treatment has led to a reduction in bleed frequency and a significant improvement in overall quality of life. No research to date has examined the nature of this improvement on treated individuals and their families.

**Aims:**

The Emi & Me study aims to capture the real‐life experience of using emicizumab for PwHi and their families.

**Methods:**

Participants were recruited through treatment centres, social media and by word of mouth. Each participant and a family member, if available, took part in a semistructured qualitative interview. All interviews were recorded, transcribed verbatim and analysed thematically. All elements of the study were reviewed by local statutory authorities and informed consent was sought from all participants.

**Results:**

Fifteen PwHi, mean age 27.2 years (range 8–63 years), most with a family member, participated in a single qualitative interview online (*n* = 13), by telephone (*n* = 1) or in person (*n* = 1). Mean time on emicizumab was 2.26 years (range 1–5 years). Six major themes emerged: bleeds; pain; treatment burden; control; freedom (for both PwHi and family members) and missed potential. Emicizumab prophylaxis has delivered significant improvements in the lives of the participants. Despite these improvements, some participants felt that their pre‐existing physical disabilities and the lack of physiotherapy provision had prevented them achieving similar improvements in their functional ability.

**Conclusion:**

This study shows that in reducing bleeds, pain and treatment burden, emicizumab had given PwHi greater control over their condition, allowing a sense of freedom they had not experienced with factor VIII or bypassing agent prophylaxis. However, for emicizumab to be truly effective, there is a need to ensure the continued availability and accessibility of robust multidisciplinary support services. Without this, it is unlikely that PwHi will realize the life‐changing potential offered either by emicizumab or any other novel treatment approach.

**Patient or Public Contribution:**

A patient participant (who did not wish to be included as an author of the paper) was involved in the design of the study protocol and interview guide.

## 1 INTRODUCTION

Prophylactic factor VIII (FVIII) replacement therapy is the standard of care for haemophilia A. It has led to significant decreases in comorbidity and improved overall quality of life for people with haemophilia (PwH).^1^, ^2^, ^3^ However, around 30% of those treated develop alloantibodies (or inhibitors) to the infused factor,^4^, ^5^ rendering treatment ineffective.

Immune tolerance therapy (ITI) aims to suppress the inhibitor, allowing the reintroduction of prophylaxis. ITI requires the infusion of large doses of FVIII, often twice daily, meaning that individuals need either good venous access (which can be problematic in young children) or a central venous access device. Up to 30% of people with haemophilia and inhibitors (PwHi) never tolerize. Bypassing agents, either FEIBA or activated factor VII (FVIIa), are used to manage breakthrough bleeds in ITI^6^ and may be used prophylactically in those with persistent inhibitors.^7^, ^8^ These agents have short half‐lives (4–7 and 2–3 h, respectively) and are less protective than FVIII prophylaxis. Both ITI and the use of bypassing agents in PwHi are burdensome due to infusion frequency and volume, and preparation and administration time.^9^

Emicizumab is a humanized monoclonal antibody that mimics the action of FVIII. It binds to activated factor IX (FIXa) and factor X (FX), normalizing the intrinsic clotting pathway and preventing spontaneous and minor traumatic bleeds.^10^ Given subcutaneously, weekly, 2‐weekly, or 4‐weekly, emicizumab was seen in clinical trials to reduce the annualized bleeding rate (ABR), decrease burden of treatment, and increase the overall quality of life for PwHi,^11^, ^12^, ^13^ It has been the standard of care treatment for PwHi in the United Kingdom since 2018.

The Emi & Me study was designed to explore the real‐world impact of emicizumab on the lives of PwHi and their families.

## 2 MATERIALS AND METHODS

### 2.1 Study design

A qualitative interview study was conducted with PwHi whose treatment had been switched to emicizumab from FVIII, FEIBA or recombinant FVIIa. Interviews were carried out by S. F. and K. K. between 1 August 2020 and 31 January 2021.

The interviews followed an interview guide (see Table 1) based on a review of the literature and the experience of the study team and a patient representative. Questions addressed the individual's haemophilia and treatment history, decision‐making, the process of changing treatment, their understanding of and any concerns about their new treatment and their experience of switching, and thoughts on their own future and that of haemophilia treatment.

**Table 1:** Interview guide

Questions	Prompts
*Part 1: People with haemophilia*	
What is your name … and how old are you?	
Can you tell me about your haemophilia—when were you diagnosed? When did you get an inhibitor? What treatment (if any) did you have for the inhibitor? What treatment were you most recently on (before emicizumab)?	
Can you recall how many bleeds you had in a month on your old treatment? Is that fairly typical for you or abnormal?	
How are your joints? Do you have any joints that bleed more than others? How did you manage those before and has that changed now?	
Have you ever had an operation in hospital? What was it for?	
How do you feel/what is your experience your current treatment?	How often do you take your treatment? What time of day do you usually take it? o Dose/when started/any change/how decided/(trial/EAMS) why?
	Why do you treat at that time of day?
	How active are you on a day‐to‐day basis? Do you do any sports?
	How have you adjusted to less frequent treatment?
	What is it like to do subcutaneous injections?—Who taught you to do them? What was it like?
	How do you record your treatment? (Haemtrack, etc.) Do you do that on paper or using the app?
What impact does emicizumab treatment have on your life?	Imagine a scale of 1–6, where 1 is no impact and 6 is the highest impact. How much of an impact on your life does your treatment represent? o Now vs. before?
	Is there anything you would like to change about your treatment?
	Have you had any bleeds since using emicizumab?
	o If yes, where were they? When did they happen? Do you know what caused them? What action did you take?
	Have you noticed any bruising at your injection site?
What about pain? Do you have any pain now? Again, imagine a scale of 1–6, where 1 is very little pain and 6 is the worst pain. How bad is that pain?	How has it been over the past month (do you get pain every day, is it joint related, is it haemophilia related)?
	What did you usually do in the past when you experience pain related to your haemophilia?—Is it different now you are on emicizumab?
	How does arthritic pain differ from the pain of a bleed (if applicable—probably not to child participants)?
	Is the pain different on emicizumab?
Have you missed a dose of treatment or been late giving it?	If you do miss a dose, what do you do about it?
	On balance, how easy is it to remember doses when given once week/fortnight/month?
How did you feel about switching to emicizumab?	Do you know how emicizumab works?
	When and how did you first hear about emicizumab?
	Who instigated the notion of switching—was it you or your clinicians?
	How often will you be treating? Why are you having that frequency? Has anyone explained pharmacokinetics? Has your doctor or nurse ever tried to explain PK to you?
	How worried are you about an injury? Why? How would you treat now vs. past?
	What impact has emicizumab had on your time—infusion time/time off school/work, etc.?
What are your hopes/expectations for emicizumab? What are your goals for the next 6 months? Do you have any concerns about it?	
Have you heard about any other treatments that might be available in the future?	Knowing how emicizumab has been for you, would you now want to consider other treatments such as gene therapy if it was available?
	How would you feel about having to go back to your last treatment (FEIBA/NovoSeven prophylaxis/on‐demand?)
*Part 2: Dyad partner participants*	
What difference has this medicine made to you/your family?	
How do you think emicizumab has impacted on you as a family/parent/carer?	
Can you give me examples of what impact this has made?	
Who does the injections?—Who taught you to do them?	
How does it compare with IV injections?—Who did those?	
Do you have any worries for the future?	
What advice would you give to others switching to emicizumab (inhibitors and haemophilia only)?	

### 2.2 Recruitment and data collection

Participants were recruited through centre referral, advertisements on social media sites, and word‐of‐mouth referrals. All participants took part in a single 1‐h interview conducted by two researchers. Children were interviewed in the presence of a parent; adults were given the option to be interviewed with a member of their family present. The initial recruitment target was 20 dyad pairs (PwHi and a family member) though recruitment could be discontinued once data saturation was achieved.

### 2.3 Analysis

Each interviewee was assigned a study number (Emi01–Emi30). All interviews were recorded and transcribed verbatim. Transcripts were thematically analysed by both researchers after each interview using inductive coding (S. F.: NVivo® for Mac; K. K.: manual coding). After coding each transcript, the researchers met to discuss, review, and refine the emergent codes to enable their exploration in subsequent interviews. On completion and analysis of the final interview, the researchers met to discuss all transcripts, further refine codes and identify themes.

### 2.4 Ethics

Written informed consent was obtained from all participants; children gave their own written assent, along with consent from a parent. All participants received a gift voucher as a ‘thank you’ for the time they gave attending interviews. Ethical approval for all elements of the study was granted by the UK Healthcare Research Authority and Research Ethics Committee (19/LO/1592).

## 3 RESULTS

### 3.1 Sample characteristics

Fifteen PwHi (nine children [8–15 years; mean 11.3 years], six adults [23–63 years; mean 51 years]) and 13 family members (10 mothers, 1 father, 2 spouses) participated in the study. Two additional family members (one mother and one spouse) withdrew from the study before interview. Five participants were given emicizumab as part of a registration study; five had started emicizumab during an early access scheme; five received the treatment after UK licensing. The mean time on emicizumab was 2.26 years (range 1–5 years) (for demographics see Table 2).

**Table 2:** Participant demographics

Participant number	Age	Treatment regimen	ABR^a^ (before switch to emicizumab)	Time on treatment (whole years)	ABR^a^ (post switch to emicizumab)	
					Traumatic	Spontaneous
Emi01	12–17	2 Weekly	7–12	3	0	0
Emi03	45–54	Weekly	19–24	5	5	0
Emi05	55–64	Weekly	>48	4	2	1
Emi07	12–17	2 Weekly	0–6	2	0	0
Emi09	8–11	Weekly	>48	3	0	2
Emi11	45–54	Weekly	19–24	2	0	0
Emi12	12–17	2 Weekly	7–12	2	0	1
Emi14	8–11	2 Weekly	0–6	1	0	0
Emi16	12–17	2 Weekly	>48	2	0	0
Emi18	18–24	Weekly	>48	2	0	0
Emi20	8–11	2 Weekly	7–12	2	2	0
Emi22	55–64	2 Weekly	19–24	1	0	0
Emi23	12–17	2 Weekly	19–24	1	8	0
Emi25	8–11	2 Weekly	19–24	2	0	0
Emi29	55–64	2 Weekly	19–24	2	0	0

Abbreviation: ABR, annualized bleeding rate.

^a^ Self‐reported figures and no corroborative evidence of bleed rates was sought.

No new codes were generated after 13 interviews. Recruitment continued for two further interviews, after which the interviewers agreed that data saturation had been achieved and recruitment should cease.

All participants took part in a single semistructured qualitative interview. Due to coronavirus restrictions, most interviews (*n* = 13) took place by video conference. One interview was conducted by telephone; one took place face to face (relevant coronavirus guidelines were followed).

Seven participants were known to at least one of the interviewers; none were known to both.

### 3.2 Overview of findings

Six major themes emerged: (1) Bleeding; (2) Pain; (3) Treatment burden; (4) Control; (5) Freedom; (6) Missed potential. These are supported with direct quotes, anonymized to study number; further quotes are given in Table 3.

**Table 3:** Further quotes in support of thematic analysis

*Theme 1: Bleeds*	
Emi01	Yeah, it's much better. I don't get any bleeds anymore.
Emi03	Yes, I mean, that head injury that I had a couple of weeks ago, I mean, that bled profusely. I mean, it really did. And I could not believe how quickly it stopped. It was remarkable. I don't think there's much that I could do.
Emi09	No, we haven't had NovoSeven since the trial. I know I've had about maybe one or two bad bleeds, but… one or two, whereas I'd used to have them pretty much weekly.
Emi10	(Emi09) is very good at identifying still if… you know, if he has… say, on Friday he twisted his ankle, he just went over on his ankle in PE, he's good at saying, ‘No, it's not a bleed’.
*Theme 2: Pain*	
Emi05	This is the other issue with Hemlibra and treatment for people like us. I personally felt, in these last three or four years, some of the pain I was having was not a bleed, it was arthritic pain. So, each patient has to take their own decision whether you want to infuse or you don't want to infuse.
Emi09	It's not as much pain, it's barely any pain that I would get normally. Maybe once in a while I might get a little tingle, but other than that it's perfect, really.
Emi10	We know he walked around in pain a lot, basically.
Emi11	I'm not great at taking pain relief. I've always been a kind of mind‐over‐matter type.
Emi18	And it's definitely made a big difference in my level of discomfort with my ankle. I still have some, but that's just part and parcel with… what 23 years' worth of bleeds into a single joint. But it's improved since going onto emi and being able to do the physio.
*Theme 3: Burden of treatment*	
Emi03	And subcutaneous injection once a week is nothing. It's not an inconvenience at all.
Emi13	It's much easier now with Hemlibra; we used to obviously lug all this other stuff around.
Emi18	I was just absolutely thrilled not to have to use a Hickman line anymore. Nothing could have made me happier than getting rid of that thing. The transition was peculiar; it was very weird to not have to go through a full rigmarole of finding a vein or accessing a Hickman line, and just being able to whip it out of the fridge, stick a needle on, draw it up and then give it. That was very weird to start with—I felt like I hadn't properly treated. But I got used to it much quicker than I was expecting to.
Emi26	I mean, it's amazing in terms of how much less time‐consuming it was. As I say, before school, if I was going to work… our mornings were just chaos, really, trying to get all of that done, get the children to school get me to work and everything.
Emi28	Yes, we do his emicizumab injections at night, so normally… whereas before we were doing injections all the time, weren't we, really. They'd take quite a long time to do
*Theme 4: Control*	
Emi01	Yeah. Yeah, I do. Yeah, I feel like I have a lot of control and I feel like I can control… more control my body, how I want to take the medication, if I want to take it in slowly or fast. Yeah. And it's more comfortable to do it.
	I feel like there's a lot of flexibility and it's much easier, as it's once every two weeks.
Emi03	Yes, it makes me feel uncomfortable, yes. Because the fact that you can administer medication and sort it out yourself, you feel like, ‘Okay, I can live in the forest as long as I have my own medication’. But then, when you know that you know you cannot do that, you're not empowered to do that, you just have to be somewhere where there's a structure or something to go to in case there's a problem. But at the same time, you know that it's very unlikely that it would be a problem, which is good.
Emi18	It's very much controlling my condition to a point that I'm more than happy with it, and the treatments that are more close to an actual, mechanical cure for the haemophilia.
*Theme 5: Freedom*	
Emi01	And the way it affects me, it stops me from doing some sports. But now it doesn't because I'm on the new treatment.
Emi02	Because the first years of his life, I always dedicated… since I graduated, I never had the opportunity to work because of all whatever was going on, up and down with his level, his inhibitor level. So, I was just looking for something that would really work so I can get on with my life, really.
Emi03	Now I can do anything I want.
Emi08	You forget how your life was like as well. You totally forget about that. It's really odd.
Emi18	Before emicizumab, because my degree required several foreign field trips, I would have had to either just not go because it would be too inconvenient, or bring with me I think 45,000 units of FEIBA, which is a pallet of FEIBA, which would be a nightmare to transport and to get through customs anywhere, because it's just vials of white powder so immediately alarm bells start ringing.
*Theme 6: Missed opportunities*	
Emi11	Somehow, I always imagined that with better treatment might come a rolling back of the years, but the things I've always wanted—things like family, career, travel the world, hobbies, things like that that I used to enjoy—all of these things are still pretty much beyond my grasp.
Emi24	He prefers it because it's less injections, but he's not more tolerant to have it. And he will not do it himself either.

### 3.3 Bleeding

All participants reported a noticeable decrease in the number of bleeds since starting emicizumab, with nine reporting no bleeds (Table 2). Six reported bleeding events, of which three were spontaneous. Of those reporting bleeds (spontaneous or traumatic), three said they had resolved more quickly than expected, and in some cases, no additional treatment with a bypassing agent had been required.

Early on in the trial, I walked into the boot lid of my car and cut my head. And usually scalp bleeds were just terrible; they would bleed and bleed and bleed and would be very difficult to control. But it stopped immediately. (Emi03)

Seven participants reported haemostatic challenges (falls, trips, grazes or surgery) for which bypassing agents had not been required.

If I fall over… like I fell over my bike a few times and I just… it will just be a bruise, that's it. Nothing else. (Emi16)

He's had two biggish injuries that would absolutely have required a lot of factor, that we watched and waited with this bruise and it's come up and then dispersed so much earlier than it ever would. (Emi21)

One mother spoke of her son undergoing a portacath removal:

I remember the surgeon afterwards speaking to me and saying… I think he was the second haemophiliac who he'd removed a port on in someone who was on emicizumab, and he just was saying how… he described the surgery as amazing as it's like there's this… there's no blood. He said it's really weird, you expect someone with haemophilia there's a certain amount of blood and things, and he said it's just incredible how… how little they bleed from it, really. (Emi26)

Three participants reported difficulty in understanding whether an injury would require treatment, or whether they should ‘wait and see’.

I just thought, ‘Oh, I think I might struggle’. And I sometimes do as well—not quite as much now, down the line—but about believing that he wouldn't need treated, that kind of thing. Like, ‘Well, surely that needs something, that was a big one’, or… that kind of thing. (Emi20)

He fell over the other week and he cracked his head, and they were going, ‘Oh…' probably thinking, ‘We'll need to go down’, and they rang the hospital and they just said, ‘Well, keep an eye on him—he should be fine’, and he was. (Emi22)

One interviewee described a period of hypervigilance, a hangover from his pre‐emicizumab days, where he was almost overly concerned that he might be about to get a bleed. He gradually became less concerned.

In the early phase of the trial, there were a few occasions when we treated with recombinant VII for what I thought were bleeds. And whether that was because the emicizumab hadn't reached the level it needed to be effective or whether it was just this hypervigilance persisting and not being entirely trusting of the emicizumab… But then there was a clear point when I thought, ‘Let's just see what happens if I don't treat—because what's the worst that can happen is I'll have a bad bleed, which I've had all my life anyway’. (Emi03)

Two participants referred to occasions before starting emicizumab on which they may have taken factor inappropriately, now believing their pain was arthritic rather than a symptom of an acute bleed.

This is the other issue with [emicizumab] and treatment for people like us. I personally felt, in these last three or four years, some of the pain I was having was not a bleed, it was arthritic pain. (Emi05)

Although her son was experiencing fewer spontaneous bleeds, one mother described continuing trauma‐related bleeds. This, combined with his dislike of the injection, meant emicizumab had not significantly improved his quality of life.

He's still bleeding when he's active. So, February to September, he had a bleed every month from falling off his bike, playfighting with his friends, anything really. And I'd say the only reason he's not had a bleed now probably since September is because of Covid restrictions as he's not been going out. He doesn't want to be on Hemlibra. He hates the injection. (Emi24)

Two participants who had been involved in registration studies referred to the risks associated with administering FEIBA as a treatment for bleeding while on emicizumab^14^ although both felt this was now less of a concern.

### 3.4 Pain

All six adult PwHi described pain as a constant part of life before emicizumab. Three said the nature of the pain they experienced had changed over the course of their life.

I think as the joints become more damaged, the bleeds become less painful. I can remember as a youngster up to my teens, bleeds would be just indescribably painful. (Emi03)

I have more aches than pains. Because like I say, my joints are wearing out, my bones are wearing out. (Emi22)

Pain relief was also an issue for these participants. Four reported an ambivalent relationship with analgesics and a reluctance to take them, partly due to side effects.

I find that the pain relief, because I've already got the exhaustion, fatigue and that side of things as much as anything, and brain fog, because of all of that, the last thing I want is another medication that's going to dampen down my brain, my thought process or my energy levels anymore. (Emi11)

Two felt they needed to keep them in reserve.

Because I'm so used to pain relief and all that sort of thing, I take [them] when I'm having severe, severe pain. (Emi06)

One said analgesia was of little use.

You can't… you can't medicate against those sorts of pains. (Emi03)

Pain was an issue raised by two mothers. One said her son had complained of constant pain in his ankles even when not in a bleed state.

I remember he just looked up and he said, ‘But my ankles always hurt, mummy’. You know, he just went, ‘They never stop hurting, mummy. They've always hurt’. (Emi10)

Another thought the level of pain experienced by children was under‐recognized:

I do wonder if they probably do feel quite a bit of discomfort but don't know that they are; the pain or discomfort is normalized. I wonder about that. I don't know. (Emi08)

Most of those reporting pain said emicizumab had made a difference to the level of pain experienced. For three adults, experiencing fewer bleeds meant a decrease in some pain.

It's improved pain levels in terms of the fact that I'm not getting bleeds, and then I'm not getting recovery periods and stuff like that. (Emi11)

One mother said her child's pain had improved after starting emicizumab.

One of the loveliest days for me—I don't know if (Emi09) would remember this, really—so in that first six months you'd be constantly still going, ‘Any aches and pains? You okay? Does anything hurt? Are you all right?’ and I remember going… I remember saying to him, ‘Is everything okay, anything ache?’ and he went, ‘No, mummy’. And I went, ‘You know how your ankle used to ache?’ and he went, ‘Mummy’, he said, ‘Nothing's hurt for such a long time’. (Emi10)

However, pain remained an issue for one participant due to damage caused by previous bleeds:

Between the arthropathy, contractures and this spasming, yes, it's quite restrictive and it's a quite painful experience. (Emi11)

### 3.5 Treatment burden

Nine participants (four PwHi, five parents) spoke of the burden of treatment before starting emicizumab.

I always knew that we needed two hours to get out—that was in my head. You know, like if somebody rang up and said, ‘Meet us up…’ I don't know, for example, ‘Meet us up the park, we're going up there in half an hour’, in the morning, say, you got a text message at eight saying, ‘We're going to be at the park at nine’, you knew that you couldn't. You'd have to go… if you hadn't done his medicine, I knew it would have to be definitely a two‐hour thing. (Emi10)

To be honest with you, the whole time is a bit of a blur. I think at the time we just went into survival mode, because even now I speak about everything we've been through and it almost feels like it didn't happen. Because it was so time‐consuming and the thought of doing that now, I just can't imagine. (Emi26)

Four participants spoke of gaining time in their lives due to shorter administration times.

I felt really happy after a while, because I realised that I didn't have to take it every single day, not take thirty minutes, just take it… And now I can just take five minutes of my time and just take my medication. It's just like a small thing that I need to do once every two weeks (Emi01)

After so long on very awkward and very clunky treatment, this is still… I still can barely believe how effective it is for such a small time commitment and such a small treatment burden. (Emi18)

One mother said her son had gained the confidence to self‐treat since starting emicizumab.

So, it's now subcut into his leg and (Emi12) now gives it himself and draws everything up and does the electronic diary, and he's really, really good at it. (Emi13)

There was recognition that while emicizumab had changed the treatment burden, it had ‘not changed the disease’ (Emi08).

All participants were aware of gene therapy as a possible cure for haemophilia, but that it might not be a possibility for them because of their inhibitor and, in the case of children, their age. Two participants said they would not want to have gene therapy even if it were available to them as emicizumab had allowed them to take back control of their condition.

I think it's as close to a cure as I'm prepared to go at the minute. Because it's very much controlling my condition to a point that I'm more than happy with it. (Emi18)

### 3.6 Control

Eleven participants (six PwHi, five family members) felt emicizumab had given them more flexibility and therefore control over their lives.

It's given me a bit more control, because it's sort of like… well, I'm not going to suffer now as much as I did earlier on in life. I feel a bit more assured, if you know what I mean. I can do something and say, ‘Well, if that doesn't go right, it's not going to hurt’. Well, it will hurt, but it's not going to hurt for two weeks as it normally did. But yes, it's made me… yes, it's good. (Emi22)

And then after maybe ten seconds it's done, and I do that once every two weeks. That's really helpful. (Emi01)

Two mothers described their child's quality of life having been limited by their condition, and that limited treatment options had led them to try what was at the time an experimental medication.

We were kind of desperate at that time […] we were looking for something that worked, because FEIBA was just unpredictable. (Emi02)

It was a leap of faith. But to be honest, our options were very limited at that time, and I just feel very, very lucky. (Emi13)

This sense of limited options and lack of control had also impacted the wider family. Eight participants (four mothers, two spouses, two PwHi) spoke of disruption to the lives of family members.

Because the first years of his life, I always dedicated… since I graduated, I never had the opportunity to work because of all whatever was going on. (Emi02)

It had a huge effect on the whole family. I became (Emi12)'s… I gave up my job, I became (Emi12)'s carer. (Emi13)

Five PwHi reported that family life had often revolved around their haemophilia.

We never used to plan our holiday with the girls. Eight out of ten times I'd have a bleed on that day, so the choice was should we continue with the holiday—and then if you continue with the holiday, you're bleeding. So, it's not just myself. And they don't enjoy the holiday. (Emi06)

For me it had a big impact, but for my sister it's had a pretty big impact as well. I think probably, even into adulthood, that impact has kind of left psychological marks. I think there's this element of feeling like you come second to… (Emi11)

### 3.7 Freedom

Eleven participants (seven PwHi, four family members) said emicizumab had given a sense of freedom—something few had experienced previously. PwHi were now able to undertake activities, both sporting and travel‐related, without fear of having a bleed.

I can go out with my friends, go on my bike when I want to. (Emi16)

There is nothing now that I can't do that I would have wanted to normally. Because I wouldn't be… I wouldn't get into boxing or play rugby, but that's because I don't like those things now, not because I'm not allowed to do them. (Emi18)

For family members, freedom included the ability to return to a career.

When he first went on emicizumab I took a new job that involved me going up to Scotland for a couple of days once a month, and I was able to do that, which… I hadn't been able to leave him. It's not something someone else can pick up, putting a needle in a vein and mixing up all that stuff. It's quite an involved process; you can't just write it on a piece of paper and ask someone else to do it. So, yes, it enabled me to take a job and allowed me to be [away] overnight. (Emi08)

Four participants (two PwHi, two family members) spoke of the whole family gaining a new sense of freedom.

You can plan much further ahead. I mean, our planning was, ‘Right, what are we doing tomorrow? Because I haven't got a bleed today’. Now, I can plan… well, we'll go on holiday in August, and with relative certainty know that you could. (Emi29)

So, I could quite happily do whatever I want; I don't have to think about if there's going to be anyone around for him. If I wanted to just go away for a week or two weeks, absolutely no question. (Emi04)

One father spoke of the change in himself as a parent since his son started emicizumab.

I was just constantly worried whenever there was a party or anything like that. And when he was out and about I was watching him like a hawk. I became Mr Health‐and‐Safety about everything. And now, like you say… I never really thought he'd be able to play football or anything. He probably told you […] he's obsessed with it now. (Emi28)

### 3.8 Missed potential

For three participants, treatment with emicizumab offered potential to bring improvements, but could not bring about an improved quality of life in and of itself. One mother hoped her son would be able to swap to an intravenous treatment (his preference) that offered the same benefits of emicizumab (specifically decreased treatment frequency).

Two participants felt emicizumab had not diminished the burden of their condition, despite reducing their bleeding rates, as potential improvements had not been realized.

Before all this lockdown scenario was I was having physio and that physio was making a great deal of difference to loosening up tendons and joints and muscles, and by loosening them up I was having days where the pain was way, way reduced compared to what I was experiencing before. And now that I'm not able to have the physio because of lockdown, even though I'm on emi, nonetheless that joint pain still keeps building up and building up again. (Emi11)

It's just got so much potential, but it's only going to achieve that potential if the rest of the system is geared up to support that, that change. (Emi11)

## 4 DISCUSSION

The registration trials reported a significant decrease in ABR in PwHi treated with emicizumab.^11^, ^12^ Participants in our study also described a reduction in the number of bleeds since starting emicizumab, although not all participants achieved zero bleeds. PwHi experience higher bleed rates than those without inhibitors because bypassing therapy prophylaxis is only partially effective, resulting in increased morbidity.^15^, ^16^ Emicizumab is the first drug that has offered PwHi a truly prophylactic treatment.

The bleeds experienced by PwHi result in pain. PwH experience acute pain due to bleeding episodes and chronic pain due to arthropathy. However, some PwH experience pain that is difficult to attribute to either active bleeds or arthropathy. In a study by Mulders et al., 18.8% of PwH experienced daily pain that correlated with both age and the presence of inhibitors.^17^ They surmized this was as a result of increasing arthropathy.

Four adults in our study noted that the nature of their pain had changed over the course of their lives. While still experiencing bleeds in adulthood, two participants believed previous joint damage made the pain of an acute bleed less severe. Three noted a further change in their experience of pain since starting on emicizumab; however, all adult PwHi continued to suffer the pain associated with arthritic changes.

Features of chronic pain can also be seen in young children without obvious signs of joint damage.^17^ Studies have shown that children with haemophilia (CwH) experience significant levels of pain, which can have longstanding negative effects, including anxiety and depression.^18^, ^19^ However, the severity, nature and duration of the pain experienced is not well understood, and our study suggests the degree of pain CwH experience is unrecognized. This may be because the pain is internalized and interpreted as a ‘normal’ part of their life rather than as pain. More research is needed to understand the pain experienced by CwH and how it can be alleviated.

Five participants, including two children, took part in the registration trials. Research suggests parents allow their children to participate in clinical trials because they hope to see a clinical benefit, they view research as a common good, wish to show gratitude to their care team and gain access to new medications.^20^, ^21^, ^22^, ^23^ The limited research on this topic in haemophilia and other lifelong diseases identified a similar list of reasons.^24^ Our study suggests desperation may also play a part. The parents of the two children who took part in registration studies felt they had little choice but for their child to participate due to the existing treatment burden on their child, themselves and their families, and the belief that unless they did so their child might never achieve their full potential.

Studies have shown that the burden of treatment on parents of CwH and inhibitors is considerable, with much time spent giving ITI and treating bleeds, often leading to distress and anger on the part of the child.^25^, ^26^, ^27^ The burden often falls on mothers in particular, although no published studies have engaged fathers to a significant degree; in our study, only one father participated. Going forward, it will be important to engage fathers in studies if we are to understand what role they play in their child's treatment and what they feel about their child's condition.

The burden of treatment seems to have been seen differently by adults in our study. Having grown up with their condition, they may have come to accept the nature of the burden. However, four adults spoke of an inability to plan their lives with any degree of certainty because of the possibility that, at any given time, they might experience a bleed. This uncertainty led to both themselves and their families feeling trapped by their condition. Many participants and their family members spoke of having more control over their lives since starting emicizumab and of having gained a new sense of freedom. For some, though, the feeling of being trapped extended now to longstanding disabilities caused by their condition. For two, while emicizumab had made a tangible difference in that they experienced fewer bleeds and less pain, the potential improvements reported by others (increased mobility, freedom to undertake sports and exercise) were unrealized because they could not access the physiotherapy and social care support that would allow them to achieve this.

### 4.1 Limitations

This study involved a small, self‐selecting sample of participants with ready access to bypassing agents and, as such, may not be representative of the entire population if PwHi or the wider population of PwH, haemophilia care teams and statutory bodies in the future.

The experience of bleeds reported here is based on the recall of individual participants rather than objective evidence. However, it does support the reduction in bleeding rates seen in the registration studies.^11^, ^12^, ^13^

Few fathers have participated in studies of this type, and in our study, we interviewed only one father. This is a common problem within haemophilia research and has been highlighted by Khair et al.^28^

## 5 CONCLUSION

Bleeding is the cardinal symptom of haemophilia, with or without an inhibitor. Treatments for haemophilia are licensed because they reduce bleeds. However, for patients, it is the impact of bleeds on everyday lives that is important. There is a growing recognition of the need for outcome measures that go beyond regulatory endpoints and which truly reflect patients' experience of haemophilia. Bleeding is the cardinal symptom of haemophilia, with or without an inhibitor. Treatments for haemophilia are licensed because they reduce bleeds. However, for patients, it is the impact of bleeds on their everyday lives that is important. There is a growing recognition of the need for outcome measures that go beyond regulatory endpoints and which truly reflect patients' experience of haemophilia and the ‘real world’ impact of their treatments.

Our study showed that in reducing bleeds, pain and treatment burden, emicizumab had given PwHi greater control over their condition, allowing a sense of freedom they had not experienced with FVIII or bypassing agent prophylaxis.

For these patients, emicizumab now offers the first truly prophylactic treatment. While inhibitor eradication with ITI is likely to remain the treatment approach of choice, it may be that emicizumab will be used with ITI to prevent bleeding.

However, for emicizumab to be truly effective, there is a need to ensure the continued availability and accessibility of robust support services. This means strengthening the multidisciplinary comprehensive care approach that has served haemophilia so well. Without this, it is unlikely that PwHi will realize the life‐changing potential offered by either emicizumab or by any other novel treatment approach.

## CONFLICT OF INTEREST

The Emi & Me project was supported with funding from Roche Products Limited and Chugai Pharma UK Ltd. Roche and Chugai conducted a factual accuracy check on the final article but any decisions to incorporate comments were made solely at the discretion of the authors

## AUTHOR CONTRIBUTIONS

*Study design*: Simon Fletcher, Kate Khair and Michael Holland. *Design of interview guide*: Simon Fletcher and Kate Khair. *Facilitation of all interviews*: Simon Fletcher and Kate Khair. *Transcription of interview recordings*: Kathryn Jenner. *Analysis of the transcripts*: Simon Fletcher and Kate Khair. *Authorship of the manuscript*: Simon Fletcher. *Approval of final manuscript*: all authors.

## Data Availability

The data that support the findings of this study are available on request from the corresponding author. The data are not publicly available due to privacy or ethical restrictions.
